# Genetic diversity of *Leptospira* in northwestern Colombia: first report of *Leptospira santarosai* as a recognised leptospirosis agent

**DOI:** 10.1590/0074-02760160245

**Published:** 2016-11-10

**Authors:** Ronald Guillermo Peláez Sanchez, Juan Álvaro Lopez, Martha María Pereira, Margarita Arboleda Naranjo, Piedad Agudelo-Flórez

**Affiliations:** 1 University of Antioquia, School of Microbiology, Medellín, Antioquia, Colombia; 2 Fundação Oswaldo Cruz, Instituto Oswaldo Cruz, Centro Colaborador da OPAS/OMS para Leptospirose, Rio de Janeiro, RJ, Brasil; 3 University CES, Antonio Roldán Betancur Hospital, Colombian Institute of Tropical Medicine, Antioquia, Colombia; 4 University CES, Faculty of Medicine, Medellín, Antioquia, Colombia

**Keywords:** leptospirosis, Leptospira, phylogenetic, MLST, monoclonal antibodies, PFGE, Colombia

## Abstract

The region of Antioquia in northeastern Colombia has the highest number of reported leptospirosis cases in the country. It also shows high seroprevalence indexes in the general population and socio-environmental conditions favourable for the transmission of the disease between humans and animals. In this study, 25 *Leptospira* isolates from Colombia’s Antioquia department were identified to the species level as *L. santarosai* (12), *L. interrogans* (9) and *L. meyeri* (4) using phylogenetic analysis of the Amidohydrolase gene. Typing at the serovar level was performed using multilocus sequence typing (MLST) and monoclonal antibodies. The serovars Canalzonae, Babudieri, Alice, Beye, and Copenhageni have been identified as causing human or animal infections in Antioquia, Colombia. The four environmental isolates were not identified to the serovar level. *L. santarosai* serovar Canalzonae and Alice were identified as new etiologic agents of human leptospirosis in Antioquia, Colombia. This paper reports species and serovars that were previously unknown in the region.

Leptospirosis is a globally distributed zoonosis that poses a major public health problem in rural and urban areas of tropical regions. This infection is attributed to direct or indirect human contact with the urine of wild, synanthropic, or domestic animals infected with *Leptospira* bacteria (Evangelista & Coburn 2010, Galloway & Levett 2010). Outbreaks of the disease in developed countries are usually associated with occupational exposure, tourism, and water sports events (CDC 1998, Morgan et al. 2002, Benschop et al. 2009, Desai et al. 2009, Stern et al. 2010). Developing countries bear the greatest burden of the disease due to the precarious living conditions of the populations exposed to the main risk factors (Bharti et al. 2003, Petrakovsky et al. 2014). While there are currently both serological and molecular classifications of *Leptospira*, there is no direct correlation between them. A given serogroup is often found in several *Leptospira* species (Cerqueira & Picardeau 2009, Adler & Moctezuma 2010, Saito et al. 2013). For instance, the 14 described serovars of the Bataviae serogroup are found in *L. interrogans* sensu stricto (five serovars), *Leptospira santarosai* (five serovars), *Leptospira kirschneri* (one serovar), *Leptospira noguchii* (two serovars) and *Leptospira borgpetersenii* (one serovar) (Cerqueira & Picardeau 2009). According to studies based on DNA/DNA hybridisation, the genus *Leptospira* consists of 21 genomic species (Saito et al. 2013). Serological studies describe over 260 pathogenic serovars, grouped into 24 serogroups, and 60 saprophytic serovars (Cerqueira & Picardeau 2009). Given the high serological and genetic diversity of *Leptospira*, it is important to identify the circulating species/serovars in order to enhance leptospirosis prevention and control strategies. For example, serodiagnostic tests could be improved by the incorporation of specific antigens for known native species. Likewise, vaccines could be developed specific to serovars circulating in different regions. The majority of Colombian studies have been limited to investigating leptospirosis outbreaks or studying seroprevalence in specific populations in certain regions of the country (Sebek et al. 1989, Epstein et al. 1995, Ochoa et al. 2000, Nájera et al. 2005, Ferro et al. 2006, Romero et al. 2010). However a few studies in Colombia have incorporated molecular and serological identification of clinical isolates by serological typing, multilocus sequence typing (MLST) and pulsed-field electrophoresis (WHO 2003, Szonyi et al. 2011, Romero-Vivas et al. 2013a, b). Since there is little information available about the genetic diversity of species and serovars in Colombia, it is important to identify the causative agents of human leptospirosis and its distribution in animal hosts and environmental sources.

## MATERIALS AND METHODS

*Source of isolates of Leptospira* - Twenty-five *Leptospira* isolates were collected during the study. Nine of these isolates were obtained from blood sampling of patients diagnosed with leptospirosis from the municipalities of Apartadó (eight) and Puente Iglesias (one). Four isolates were obtained from the kidneys and blood of capuchin monkeys (*Cebus capucinus)* that died as a result of jaundiced leptospirosis in the municipality of Barbosa. Three isolates were obtained from urine samples from dogs in the city of Medellin, which were sent to the laboratory for suspected leptospirosis, and five isolates were obtained from kidney samples from *Rattus norvegicus* captured in the city of Medellin and the municipality Turbo. Finally, four isolates were obtained from environmental water sources collected in the municipalities of Triganá and Necoclí; these water sources are used as for human consumption. All isolates were grown in liquid EMJH medium supplemented with 10% EMJH enrichment medium (Becton-Dickinson Biosciences) at a temperature of 26-30ºC. The cultures were examined by dark field microscopy weekly for three months to detect *Leptospira* growth*.*

*DNA extraction* - DNA was extracted from 1 mL of culture. The turbidity of the bacterial suspensions was adjusted to 0.5 McFarland standards. DNA extraction was performed using Wizard Kit (Promega®, USA), according to the manufacturer’s instructions for gram-negative bacteria. All experiments were performed at a concentration of 20 ng/µL DNA for isolates. Concentration and purity were determined by Nanodrop, while integrity was assessed by 1% agarose gel electrophoresis.

*Amidohydrolase gene amplification* - A 914-base pair fragment from the Amidohydrolase gene was amplified by polymerase chain reaction (PCR) using the primers F16S (GCGGATATGCCGAACAACCCG) and R16S (TCAAACGGGCTCCAGCCGCT). The reagent concentrations used for PCR standardisation were as follows: primers (0.4 μM), dNTPs (0.2 mM), buffer (1×), MgCl2 (1.5 mM), Taq polymerase (1 unit/reaction) and DNA (200 ng/μL). The final volume for each reaction was 25 μL. PCR was performed in a Perkin Elmer 9700 thermocycler. The thermal cycling profile was: one initial denaturation cycle at 94ºC for 5 min, followed by 35 cycles at 94ºC for 45 s, 64ºC for 1 min, 72ºC for 2 min and a final cycle at the extension temperature of 72ºC for 5 min.

*Species identification by phylogenetic analysis* - The Amidohydrolase genes from 19 *Leptospira* species were used as reference sequences. These were: (WP_010573489.1, WP_020772111.1, WP_003005945.1, WP_004442891.1, WP_002745964.1, WP_004768995.1, WP_010577276.1, WP_046951104.1, WP_000591986.1, WP_039948702.1, WP_020987613.1, WP_002972272.1, WP_039935685.1, WP_002977290.1, WP_015682624.1, WP_015676897.1, WP_004786049.1, WP_016546771.1, and WP_012387340.1). These sequences and those obtained from the isolates by sequencing were aligned using the ClustalX program (Larkin et al. 2007). Phylogenetic analysis was performed with using MEGA6 phylogenetic software (Tamura et al. 2011), using the Neighbour-Joining method with 1000 bootstrap replicates. Evolutionary distances were computed using the parametric method Kimura-2.

*Serovar identification by MLST* - Molecular identification of the *Leptospira* isolates was performed using MLST, as described by (Boonsilp et al. 2013). In brief, seven genes that are constitutively expressed in *Leptospira* (glmU, pntA, sucA, tpiA, pfkB, mreA, and CaiB) were amplified and sequenced. Subsequently, these sequences were queried against a database (http://leptospira.mlst.net/) to determine their allelic profile and phylogenetic relationship to the reference strains previously included in the database.

*Serovar identification by pulsed field gel electrophoresis* - Pulsed field gel electrophoresis (PFGE) was performed using the *NotI* restriction enzyme to generate fingerprint patterns, as previously described by (Machry et al. 2010). Pulse marker 0.5-1000 kb (SIGMA) was used as a size standard. Fingerprint patterns were analysed using GelCompar II (Gel electrophoresis software, Applied Maths). Dendrograms were created from UPGMA cluster analyses based on the Dice band-based coefficient. Band comparison settings of 1.5% optimisation and 1% position tolerance were used. Fingerprint patterns for the clinical isolates were queried against a library of 19 reference serovars based on mean similarity. Those with fingerprint patterns matching reference patterns in the library were identified to serovar level (Tenover et al. 1995).

*Serovar identification by monoclonal antibodies* - Monoclonal antibodies (F70C14, F70C24 and F70C12) were used to differentiate the Icterohaemorrhagiae and Copenhageni serovars. The reference immune sera were provided by the Royal Tropical Institute (KIT) in the Netherlands. The reference strains *L. interrogans* (serogroup Icterohaemorrhagiae, serovar Icterohaemorrhagiae, RGA) and *L. interrogans* (serogroup Icterohaemorrhagiae, serovar Copenhageni, M20) were used as controls for the tests. Serial dilutions (1/20) were performed to determine antibody titres (Tenover et al. 1995).

*Clinical, laboratory and epidemiological data* - Five patients enrolled in this study were subjected to physical examinations. Peripheral blood samples were collected in the hospitals where these patients were admitted, and sent to the Colombian Institute of Tropical Medicine for laboratory tests (Indirect Immuno-fluorescence test (IIFT), microscopic agglutination test (MAT) and blood culture. Finally, the patients’ possible exposure to potential risk factors was evaluated with an epidemiological survey.

## RESULTS

**Fig. 1:**
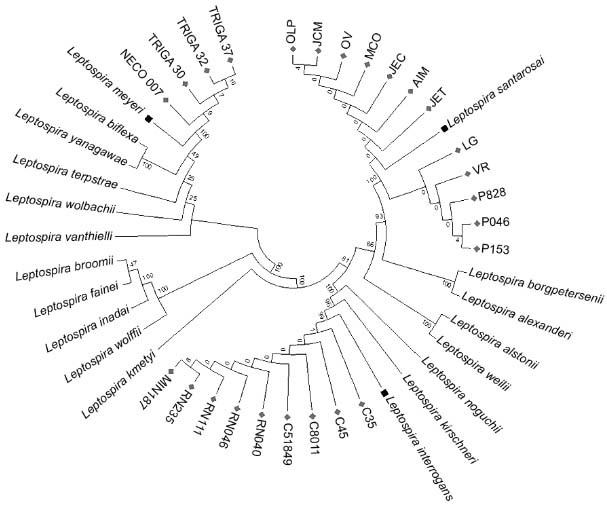
: species identification by phylogenetic analysis of the Amidohydrolase gene. A diamond and square represent isolates of *Leptospira* and the reference species genetically related to isolates, respectively.

*Species identification by phylogenetic analysis* - The species patterns were divided into three main branches according to their pathogenicity status (pathogenic, intermediate, and saprophytic). Branch support values ranged between 20-100%, permitting the identification of 19 *Leptospira* species. Isolates from humans, dogs, rodents, and capuchin monkeys (*C. capucinus*) clustered with the pathogenic species. Isolates from humans and canines were identified as *L. santarosai (*branch support values of 100%), while isolates from capuchin monkeys and rodents were identified as *L. interrogans (*branch support values of 99%). Finally, isolates from environmental water sources were grouped with saprophytic species. These were identified as *L. meyeri (*branch support values of 100%, Fig. 1, Table V).

**TABLE V:** Species level identification of twenty-five *Leptospira* isolates by phylogenetic analysis and identification to the serovar level from twenty-one *Leptospira* isolates by pulsed field gel electrophoresis (PFGE), multilocus sequence typing (MLST), and monoclonal antibodies

Code	Isolate	Source	Origin	Phylogenetic	MLST	Monoclonal antibody
1	JET	human	Apartadó	*L. santarosai*	Canalzonae	
2	AIM	human	Puente Iglesias	*L. santarosai*	Canalzonae	
3	JEC	human	Apartadó	*L. santarosai*	Canalzonae	
4	MCO	human	Apartadó	*L. santarosai*	Alice	
5	OV	human	Apartadó	*L. santarosai*	Alice	
6	OLP	human	Apartadó	*L. santarosai*	Alice	
7	JCM	human	Apartadó	*L. santarosai*	Canalzonae	
8	LG	human	Apartadó	*L. santarosai*	Alice	
9	VR	human	Apartadó	*L. santarosai*	Alice	
10	C35	*C. capucinus*	Barbosa	*L. interrogans*	unidentified	Copenhageni
11	C45	*C. capucinus*	Barbosa	*L. interrogans*	unidentified	Copenhageni
12	C8011	*C. capucinus*	Barbosa	*L. interrogans*	unidentified	Copenhageni
13	C51849	*C. capucinus*	Barbosa	*L. interrogans*	unidentified	Copenhageni
14	P828	canis	Medellín	*L. santarosai*	Beye	
15	P046	canis	Medellín	*L. santarosai*	Beye	
16	P153	canis	Medellín	*L. santarosai*	Babudieri	
17	RN040	*R. norvergicus*	Turbo	*L. interrogans*	unidentified	Copenhageni
18	RN046	*R. norvergicus*	Turbo	*L. interrogans*	unidentified	Copenhageni
19	RN111	*R. norvergicus*	Turbo	*L. interrogans*	unidentified	Copenhageni
20	RN235	*R. norvergicus*	Turbo	*L. interrogans*	unidentified	Copenhageni
21	MIN 187	*R. norvergicus*	Medellín	*L. interrogans*	unidentified	Copenhageni
22	NECO 007	water	Necoclí	*L. meyeri*		
23	TRIGA 30	water	Triganá	*L. meyeri*		
24	TRIGA 32	water	Triganá	*L. meyeri*		
25	TRIGA 37	water	Triganá	*L. meyeri*		

*Serovar identification by MLST* - MLST methodology was used to verify correct species identification results from the phylogenetic analysis of the Amidohydrolase gene, and to identify twelve isolates to the serovar level. Five isolates obtained from humans were identified as the *L. santarosai* serovar Alice, and four human isolates were identified as the *L. santarosai* serovar Canalzonae. Two isolates from canines were identified as the *L. santarosai* serovar Beye, and one canine isolate was identified as the *L. santarosai* serovar Babudieri. The four isolates from capuchin monkeys and five isolates from rodents could not be identified to the serovar level. There was a 100% correlation between the results obtained by MLST and those obtained by the phylogenetic identification method (Tables I, V). The environmental isolates were not identified to the serovar level by MLST, due to the absence of allelic profiles in the MLST database.

**TABLE I:** Identification to serovar level of 12 *Leptospira* isolates by multilocus sequence typing (MLST) methodology

Code	Species	Serogroup	Serovar	ST	Allelic profile	Country	Source
CZ188	*L. santarosai*	Grippotyphosa	Canalzonae	176	41, 53, 52, 49, 57, 43, 43	Panamá	spiny rat
1	*L. santarosai*	Grippotyphosa	Canalzonae	176	41, 53, 52, 49, 57, 43, 43	Colombia	human
2	*L. santarosai*	Grippotyphosa	Canalzonae	176	41, 53, 52, 49, 57, 43, 43	Colombia	human
3	*L. santarosai*	Grippotyphosa	Canalzonae	176	41, 53, 52, 49, 57, 43, 43	Colombia	human
7	*L. santarosai*	Grippotyphosa	Canalzonae	176	41, 53, 52, 49, 57, 43, 43	Colombia	human
1537U	*L. santarosai*	Shermani	Babudieri	172	40, 48, 47, 47, 52, 42, 42	Perú	pig
16	*L. santarosai*	Shermani	Babudieri	172	40, 48, 47, 47, 52, 42, 42	Colombia	canine
CI40	*L. santarosai*	Autumnalis	Alice	173	40, 53, 51, 48, 56, 44, 43	Sri Lanka	human
4	*L. santarosai*	Autumnalis	Alice	173	40, 53, 51, 48, 56, 44, 43	Colombia	human
5	*L. santarosai*	Autumnalis	Alice	173	40, 53, 51, 48, 56, 44, 43	Colombia	human
6	*L. santarosai*	Autumnalis	Alice	173	40, 53, 51, 48, 56, 44, 43	Colombia	human
8	*L. santarosai*	Autumnalis	Alice	173	40, 53, 51, 48, 56, 44, 43	Colombia	human
9	*L. santarosai*	Autumnalis	Alice	173	40, 53, 51, 48, 56, 44, 43	Colombia	human
Alice	L. santarosai	Mini	Beye	178	43, 51, 47, 48, 55, 45, 43	Panamá	spiny rat
14	*L. santarosai*	Mini	Beye	178	43, 51, 47, 48, 55, 45, 43	Colombia	canine
15	*L. santarosai*	Mini	Beye	178	43, 51, 47, 48, 55, 45, 43	Colombia	canine

**Fig. 2:**
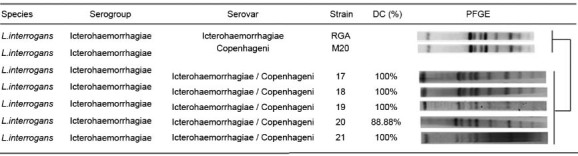
: identification of five isolates from rodents by pulsed field gel electrophoresis (PFGE). The genomic restriction profiles were consistent with the profiles corresponding to serovar Icterohaemorrhagiae or serovar Copenhageni.

*Pulsed-field gel electrophoresis standardisation* - Five isolates from rodents were identified by PFGE, as *L. interrogans* serovar Icterohaemorrhagiae or Copenhageni (Fig. 2, Table V). There was a 100% correlation between the results obtained by PFGE and those obtained by MLST. Environmental, human, and canine isolates were not identified to the serovar level by PFGE, due to the insufficient number of cleavage events by the *NotI* enzyme on the genomes of these species.

*Serovar identification by monoclonal antibodies* - Isolates from dogs and rodents that could not be identified to the serovar level using MLST or PFGE were identified using monoclonal antibodies as belonging to the Copenhageni serovar. The nine isolates had positive agglutination reactions with the monoclonal antibodies F70C24 and F89C12, with titres between 1/640 and 1/5120. They had no agglutination reaction with the monoclonal antibody F70C14, which is specific to the serovar Icterohaemorrhagiae (Tables II, V).

**TABLE II:** Identification of nine isolates from rodents and capuchin monkeys (*Cebus capucinus*) by monoclonal antibodies. The agglutination reactions identified nine isolates belonging to serovar Copenhageni

Monoclonal antibody	Serogroup-specific	Serovar-specific	
F70C14	Icterohaemorrhagiae	Icterohaemorrhagiae	
F70C24	Icterohaemorrhagiae	Copenhageni	
F89C12	Icterohaemorrhagiae	No Icterohaemorrhagiae	

Antigens	Monoclonal antibody		

	F70C14 title	F70C24 title	F89C12 title

RGA (serovar Icterohaemorrhagiae)	20,480	negative	negative
M20 (serovar Copenhageni)	negative	10,420	2,560
(10) C35	negative	20,480	2,560
(11) C45	negative	20,480	640
(12) C8011	negative	20,480	5,120
(13) C51849	negative	20,480	640
(17) RN040	negative	20,480	2,560
(18) RN046	negative	20,480	1,280
(19) RN111	negative	20,480	640
(20) RN235	negative	20,480	5,120
(21) MIN187	negative	20,480	640

*Clinical, laboratory and epidemiological patient data* - The clinical histories and laboratory test results of five patients are shown in (Table III), according to random patient numbering (P1-P5) and the respective infecting serovar. The epidemiological features related to the main risk factors and the infecting serovar are shown in (Table IV). The *L. santarosai* serovar Canalzonae was found to be the causative agent of leptospirosis in three patients. One patient showed clinical signs and symptoms characteristic of Weil’s disease. The *L. santarosai* serovar Alice was found to be the causative agent of leptospirosis in two patients. The patients infected with serovar Canalzonae came from urban areas and the patients infected with the serovar Alice came from rural areas.

**TABLE III:** Epidemiological data from five patients diagnosed with leptospirosis

Epidemiological Data	Patient 1 Serovar Canalzonae	Patient 2 Serovar Canalzonae	Patient 3 Serovar Canalzonae	Patient 4 Serovar Alice	Patient 5 Serovar Alice
Age	7	13	26	39	48
Sex	Male	Male	Male	Male	Female
Occupation	Student	Student	Employee	Farmer	Housewife
Origin	Urban area	Urban area	Urban area	Rural area	Rural area
Aqueduct	Yes	Yes	No	No	No
Sewerage	Yes	Yes	No	No	No
Drinking water	Yes	Yes	No	No	No
Artesian well	No	No	Yes	No	No
Contact with mice	Yes	Yes	Yes	Yes	Yes
Contact with dogs	No	No	Yes	Yes	Yes
Contact with other mammals	No	No	Yes	Yes	Yes
Bathing in streams	No	Yes	No	No	No
Nearby farms flooded	Yes	No	Yes	Yes	Yes

**TABLE IV:** Clinical and laboratory data from five patients diagnosed with leptospirosis

Patient / Infecting Serovar	Clinical Presentation and Outcomes	Laboratory Tests	Serological Tests
P1 / Canalzonae	Biphasic fever, chills, severe headache, frequent coughing, nausea, inappetence, generalized erythema, tachycardia (HR 120). Positive tourniquet test. The clinical course featured a mild anicteric form.	No records	IIFT positive. Seroconvert in two serum samples. Negative to 1:80 for IgM and IgG antibodies.
P2 / Canalzonae	Fever, headache, malaise, ocular itching, conjunctival, persistent cough. Proof of the tourniquet (+). The patient responded well to treatment with Doxycycline. The clinical course featured a mild anicteric form.	leukocytosis PMN: 88.4%; CRP 48 mg / dL	IIFT positive. Seroconvert in two serum samples. Negative to 1:80 for IgM and IgG antibodies.
P3 / Canalzonae	Fever, chills, jaundice, general malaise, headache, myalgia, arthralgia, nausea, vomiting, diarrhea, abdominal pain, cough, nasal congestion, red eyes, gingival bleeding, hepatomegaly. The patient responded well to supportive care and to treatment with Ceftriaxone. The clinical course featured the classical Weil’s syndrome.	Leukocytosis (16410); PMN: 83.4%; CRP: 203,55 mg / dL, BT: 4.71; Hematuria (200/AP); Proteinuria (100 /) Hypocalcemia (Potassium: 2.86 MEQ / L).	IIFT positive. Seroconvert in two serum samples. Negative to 1:80 for IgM and IgG antibodies. MAT positive, titer of 1:400 with serovar Hardjo
P4 / Alice	Fever, chills, general malaise, nausea, headache, retro-orbital pain, myalgia, arthralgia, back pain, tachycardia (HR 105). The patient progressed satisfactorily without antibiotic treatment during the acute phase of the disease. The clinical course featured a mild anicteric form.	CRP: 96	IIFT positive. Seroconvert in two serum samples. Negative to 1:80 for IgM and IgG antibodies. MAT positive, titer of 1:200 with the serovar Hardjo.
P5 / Alice	Fever, chills, malaise, headache, myalgia, arthralgia, back pain, retro-orbital pain, nausea, diarrhea. The patient progressed satisfactorily during their hospital management. No records of antibiotic treatment. The clinical course featured a mild anicteric form with hepatic changes.	CRP: 229,5 GPT: 147,6	IIFT positive with seroconvert titles from 0 (negative) to 1:160 for IgM antibodies and seroconvert from 1:20 to 1:160 for IgG antibodies.

## DISCUSSION

Characterisation of the genetic diversity of *Leptospira* species and serovars distributed in the environment, including in both reservoirs and accidental hosts, is highly advantageous for improvement of serological diagnosis, immunoprophylaxis, prevention, and control of the disease. The incorporation of native serovar antigens would increase the sensitivity of serological tests, and enable the development of more effective vaccines that target specific serovars circulating in given regions. In addition, the identification of disease reservoirs further aids in the development of prevention and control strategies, such as those aiming to minimise the dispersion of *Leptospira* bacteria in the environment and decreasing human contact with infected animals.

Human and canine isolates were identified as *L. santarosai*. This is the first report documenting the Canalzonae, Babudieri, Alice and Beye serovars as causes of human or animal leptospirosis in Antioquia, Colombia. However, this species is a known causative agent of human leptospirosis in Costa Rica and the French West Indies (Valverde et al. 2008, Bourhy et al. 2013). Previous studies in Peru also suggest the ubiquity of *L. santarosai* and the possibility of animal hosts. *L. santarosai* has been isolated from rats, marsupials, and humans and has also been found in samples taken from the surrounding environment (Rivera et al. 2012). While Brazil and Mexico have not reported cases of human leptospirosis yet, this species has been found to infect buffalo and cattle in these countries (Vasconcellos et al. 2001, Carmona-Gasca et al. 2011).

The sensitivity of the MAT may be improved by including local strains or serovars, mainly those belonging to the species *L. santarosai*. Likewise, more effective vaccines could be developed by considering these serovars in their antigenic composition. The isolates from capuchin monkeys and rodents were identified as *L. interrogans*; this species is the major causative agent of leptospirosis worldwide (Levett et al. 2006). In Colombia, *L. interrogans* has been found to infect capuchin monkeys, rodents and pigs (Szonyi et al. 2011, Romero-Vivas et al. 2013a, b).

Copenhageni and Icterohaemorrhagiae serovars are normally included in serological tests and vaccines. Since molecular identification by MLST and PFGE could not differentiate between Copenhageni and Icterohaemorrhagiae serovars, it was necessary to use monoclonal antibodies to differentiate between these serovars. The results indicate that rodent and capuchin monkey isolates belonged to the Copenhageni serovar (Table II).

The isolates from environmental water sources were identified as *L. meyeri* species, which is difficult to taxonomically classify due to it pathogenic serovars, such as Sophia, and saprophytic serovars, such as Semaranga. Other *L. meyeri* serovars, for example Perameles and Ranarum, can be found in both pathogenic and saprophytic subgroups (Kmety & Dikken 1993). Therefore, it is important to control the presence of this species in environmental water sources, due to its potential pathogenicity. The identification of these species in environmental water samples is also indicative that certain pathogenic strains may be able to temporarily survive outside of their animal hosts. The saprophytic *L. meyeri* species has not been reported in Colombia.

For epidemiological purposes, it is important to note that the three isolates identified as serovar Canalzonae came from patients living in urban areas, while isolates of serovar Alice infected patients living in rural areas. The common epidemiological factor reported for all five patients was contact with rodents in their homes (Table III). In terms of clinical characteristics, two cases (P1 and P2) were paediatric patients (under 15 years) infected with the serovar Canalzonae. One presented a biphasic course and increased indicators of acute phase disease (leucocytosis and CRP). Both patients exhibited haemorrhagic manifestations, which were evaluated by the tourniquet test. They had mild forms of leptospirosis, which were treated with amoxicillin and doxycycline. Patient P3, also infected by the serovar Canalzonae, presented clinical signs and symptoms matching Weil’s syndrome (Table IV). The patients infected with the Alice serovar (P4 and P5) also presented with acute febrile illness with systemic manifestations. One of them required hospital management for hepatitis, and had increased ALT (160.2 mg/dL), and increased total bilirubin (2.29 mg/dL) at the expense of direct bilirubin (1.8 mg/dL). Other laboratory examinations showed a tendency toward leucopoenia, with mild thrombocytopaenia and enhanced CRP (227.5 mg/dL) (Table IV). The symptomatology of patients infected with the Canalzonae and Alice serovars reflects the ability of *L. santarosai* to produce clinical symptoms ranging from mild to severe, including Weil’s syndrome and liver failure. Although other bacterial diseases can also cause increased acute phase proteins, this particular symptom may help in clinical management of the disease, mainly by aiding in the differentiation of leptospirosis from malaria, dengue, or other viral infections in patients with acute febrile disease. The interaction between animals and human and its effect on public health is an open field for research. It is important to establish the main reservoirs of the disease in order to evaluate the magnitude of leptospirosis in Colombia.
